# RET Copy Number Alteration in Medullary Thyroid Cancer Is a Rare Event Correlated with RET Somatic Mutations and High Allelic Frequency

**DOI:** 10.3390/genes12010035

**Published:** 2020-12-29

**Authors:** Teresa Ramone, Chiara Mulè, Raffaele Ciampi, Valeria Bottici, Virginia Cappagli, Alessandro Prete, Antonio Matrone, Paolo Piaggi, Liborio Torregrossa, Fulvio Basolo, Rossella Elisei, Cristina Romei

**Affiliations:** 1 Endocrine Unit, Department of Clinical and Experimental Medicine, University of Pisa, 56124 Pisa, Italy; teresa.ramone@hotmail.it (T.R.); chiaramule93@gmail.com (C.M.); raffaele.ciampi@unipi.it (R.C.); valeriabottici@gmail.com (V.B.); virginiacapp@gmail.com (V.C.); alessandro.prete22@gmail.com (A.P.); anto.matrone@yahoo.com (A.M.); paolo.piaggi@gmail.com (P.P.); cristina.romei@unipi.it (C.R.); 2 Department of Surgical, Medical, Molecular Pathology, University of Pisa, 56124 Pisa, Italy; libo.torregrossa@gmail.com (L.T.); fulvio.basolo@med.unipi.it (F.B.)

**Keywords:** medullary thyroid cancer, *RET*, copy number variation, MLPA

## Abstract

Copy number variations (CNV) of the *RET* gene have been described in 30% of Medullary Thyroid Cancer (MTC), but no information is available about their role in this tumor. This study was designed to clarify *RET* gene CNV prevalence and their potential role in MTC development. *RET* gene CNV were analyzed in 158 sporadic MTC cases using the ION Reporter Software (i.e., in silico analysis) while the multiplex ligation-dependent probe amplification assay (i.e., in vitro analysis) technique was performed in 78 MTC cases. We identified three categories of *RET* ploidy: 137 in 158 (86.7%) cases were diploid and 21 in 158 (13.3%) were aneuploid. Among the aneuploid cases, five out of 21 (23.8%) showed an allelic deletion while 16 out of 21 (76.2%) had an allelic amplification. The prevalence of amplified or deleted *RET* gene cases (aneuploid) was higher in *RET* positive tumors. Aneuploid cases also showed a higher allelic frequency of the *RET* driver mutation. The prevalence of patients with metastatic disease was higher in the group of aneuploid cases while the higher prevalence of disease-free patients was observed in diploid tumors. A statistically significant difference was found when comparing the ploidy status and mortality. *RET* gene CNVs are rare events in sporadic MTC and are associated with *RET* somatic mutation, suggesting that they could not be a driver mechanism of tumoral transformation per se. Finally, we found a positive correlation between *RET* gene CNV and a worse clinical outcome.

## 1. Introduction

Thyroid cancer (TC) is the most common malignancy of the endocrine system, accounting for about 3% of all cancers diagnosed annually worldwide (https://seer.cancer.gov/statfacts/html/thyro.html). Most thyroid tumors have epithelial origin, such as papillary (PTC), follicular (FTC), and anaplastic cancer (ATC), while medullary thyroid cancer (MTC) arises from parafollicular C cells [1]. MTC can occur both as hereditary and sporadic: germline *RET* mutations are present in 95–98% of hereditary cases [2] while they are present in about 50–60% of sporadic cases [3]. Moreover, in sporadic MTC RAS point mutations occur in about 25% of cases and they are mutually exclusive with *RET*. So far, RAS is the only *RET* alternative driver oncogene in sporadic MTC and 20–25% of these tumors are still “orphan” of driver mutations despite the deep analysis recently performed with either targeted next-generation sequencing (NGS) or with whole-genome sequencing (WGS) [4,5,6,7].

With the belief that *RET* might be the major player in the pathogenesis of MTC we previously explored the possible role of a differential expression of the two *RET* isoforms [8]. We found that not mutated MTC samples had a significantly higher expression of *RET51* with respect to *RET9* isoform while no difference was observed in the 2 *RET* isoform expression levels in *RAS* or *RET* positive cases. Although not proven, the overexpression of *RET51* in *RET* negative cases might be an alternative method of *RET* activation. Copy number variations (CNV) of the *RET* gene have been described in about 30% of MTC, either chromosome 10 aneuploidy or *RET* gene amplification [9]. It is known that the role of CNV is different among different tumors. For PTC, it has emerged that somatic CNV has been identified in cases not harboring driver mutations or fusions, thus suggesting a potential role of CNV as a driver [10]. So far, no similar data are available in MTC except for our previous study [9] in which *RET* gene CNV were associated mainly with *RET* mutation thus countered the hypothesis of a driver role. However, this study was performed before the introduction of advanced molecular testing and some data could have been missed. In the present study, we analyzed the *RET* gene CNV in a large series of sporadic MTC by using the ION Reporter Software (i.e., in silico analysis) and using the multiplex ligation dependent probe amplification assay (MLPA) technique (i.e., in vitro analysis) to better clarify their prevalence and potential role in MTC development.

## 2. Materials and Methods

### 2.1. Study Group

A total of 158 sporadic MTC patients are included in this study. Tumoral tissues were collected at the time of surgery at the Department of Surgery of the University of Pisa (Italy). MTC diagnosis was confirmed at histology. All patients were diagnosed and followed up at the Unit of Endocrinology of the Department of Clinical and Experimental Medicine of the University Hospital of Pisa. Altogether, 130 out of 158 (82.3%) samples were primary tumors, 20 out of 158 (12.6%) were lymph-node metastases and 8 out of 158 (5.1%) were tumor recurrences. An informed consent form for *RET* genetic screening and other clinical procedures was signed by all patients. The present study was approved by the Institutional Review Board and by the “Comitato Etico Regionale per la Sperimentazione Clinica della Regione Toscana” Prot n 6714, 05/02/2019. The study follows the rules of the Declaration of Helsinki.

The somatic mutation profile of all cases was previously identified using an NGS approach. Briefly, DNA was extracted from fresh tumoral tissues (*n* = 129) and formalin-fixed paraffin-embedded (FFPE) tissues (*n* = 29), using an automated method Maxwell16^®^ (Promega, Madison, WI, USA). Ion S5 targeted sequencing NGS method using a custom panel designed using the AmpliSeq Designer tool was applied. Details have been previously reported [5].

### 2.2. Methods

In silico CNV data analysis: The Ion Reporter™ Software was used to extrapolate *RET* gene CNV using, as requested by the system, the genomic DNA obtained from 20 normal male subjects as a baseline. The CNV ratio call thresholds were derived empirically by comparing data of MTC samples with the ones obtained in the baseline and harboring a normal *RET* CNV status. CNV ratio measured CNV gene locus coverage relative to coverage diploid samples. This analysis was conducted on the entire series of 158 sporadic MTC.

MLPA in vitro assay: MLPA experiments were performed in a subgroup of 78 cases using the commercial kit SALSA MLPA P169 HIRSCHSPRUNG PROBEMIX (MRC-Holland, Amsterdam, The Netherlands). This kit can detect CNV of *ZEB2*, *EDN3*, *GDNF*, and *RET* genes. The experiments were performed on DNA extracted from tumor samples following the manufacturer’s instructions. As a reference we used DNA obtained from the thyroid tissue of healthy subjects; a negative control (no-template control) was also included. All data have been analyzed using the Coffalyser.Net software (MRC-Holland, Amsterdam, The Netherlands). For each probe we obtained the Dosage Quozient value (DQ): cases were classified to have a *RET* gene deletion when DQ was less than 0.8; a *RET* gene diploidy when DQ was >0.8 but <1.3; a *RET* gene amplification when DQ was >1.3.

Comparison of the “in silico” and “in vitro” analyses: The CNV ratio values generated by the ION Reporter were compared with the MLPA DQ values to classify the samples as deleted, amplified, or diploid. MLPA DQ values were matched with the CNV ratio values to identify *RET* diploid, amplified, and deleted cases. By using the ION Reporter software, we obtained CNV values of 158 MTC samples, including the 78 cases analyzed by MLPA.

Clinical and genetic data collection: Clinical data mainly regarding the outcome of patients at the time of the study have been collected at the Unit of Endocrinology of the Department of Clinical and Experimental Medicine of the University Hospital of Pisa where all patients were followed up. The genetic profile of all samples was also available since they were previously investigated [5].

Statistical analysis: Statistical analysis was performed with the StatView 5.0 program. The correlation between the presence of the *RET* somatic mutation and *RET* CNV categories was analyzed by the Chi-squared test. The differences in the outcome vs. mutational status categories, Variant allelic frequency (VAF) value, and CNV status were evaluated by 1-way ANOVA and unpaired Student’s t-test. Data were normally distributed according to the Shapiro–Wilk test. Differences were considered statistically significant when the P-value was less than 0.05. Survival curves was performed with GraphPad Prism 9.0 using the Gehan–Breslow–Wilcoxon test.

## 3. Results

### 3.1. Analysis of the RET Copy Number

**Figure 1:**
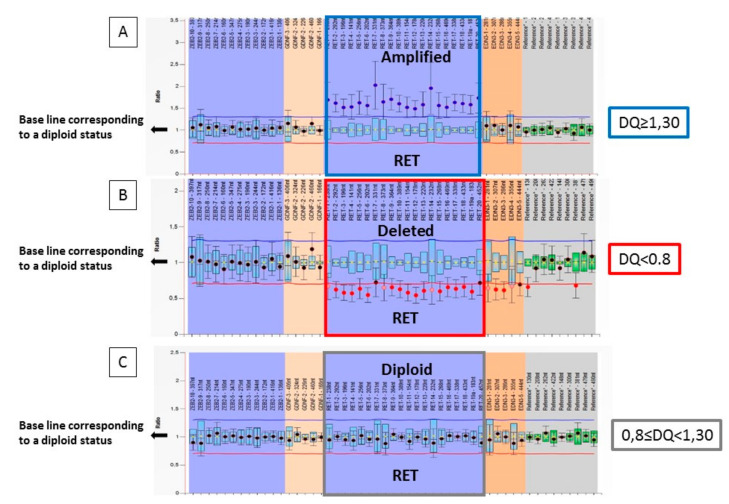
Graphic representation of the *RET* ploidy analyzed by Multiple Ligation Probe Amplification (MLPA) technique: (**A**) Probe signals are above the baseline indicating *RET* gene amplification; (**B**) Probe signals are below the baseline indicating *RET* gene deletion; (**C**) Probe signals are within the baseline indicating the presence of 2 copies of the *RET* gene.

MLPA assay. Seventy-eight MTC samples were analyzed by MLPA to detect CNV of the RET gene. Figure 1 shows three representative cases of a RET gene amplification (panel A), of a RET deletion (panel B), and a diploid case (panel C). On the basis of the DQ value, three in 78 (3.8%) cases showed a deletion of the RET gene, 66 in 78 (84.6%) cases appeared to be diploid and, finally, nine in 78 (11.6%) cases showed an amplification of the RET gene (Table 1).

**Figure 2:**
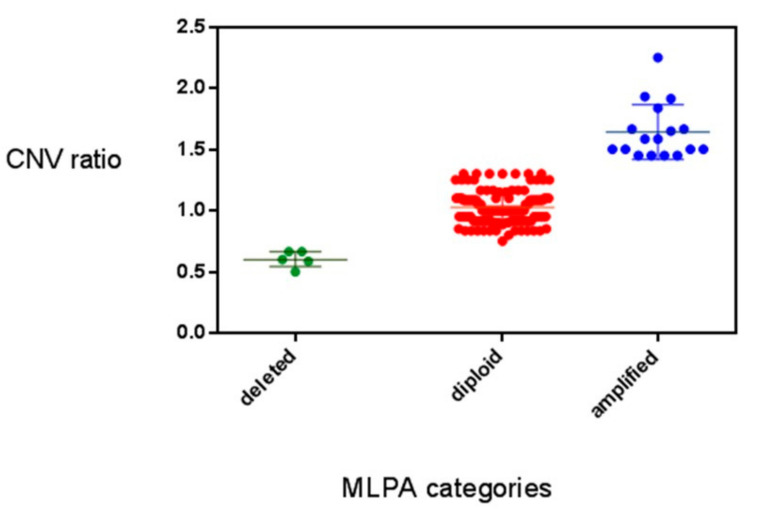
Comparison between MLPA and Copy Number Variation (CNV) values showing 3 different CNV categories.

ION Reporter. The results of the “in silico” analysis showed a series of values corresponding to each sample with a CNV ratio varying from 0.5 to 2.25. However, we observed that there were values very similar (i.e., decimals of differences) that were difficult to be assigned to the deleted, diploid, or amplified status. To better understand the meaning of these values we compared the results of 78 cases analyzed by MLPA with their corresponding in silico values. As shown in Figure 2, we identified three well-defined categories: a CNV in silico ratio >1.4 that corresponded to a *RET* gene amplification as defined by MLPA; a CNV in silico ratio <0.7 that corresponded to a *RET* gene deletion and a CNV in silico ratio between 0.7 and 1.4 that corresponded to the *RET* diploid status according to MLPA. This correspondence was so precise (Figure 2) that we decided to use the same cut-off also for the other 80 cases for which the MLPA could not be done for technical issues.

According to this definition, we obtained that five in 158 (3.2%) cases could be considered to have a *RET* gene deletion, 137 in 158 (86.7%) cases could be considered to be diploid and 16 in 158 (10.7%) cases could be considered to have a *RET* gene amplification (Table 2).

### 3.2. Correlation between RET Gene CNV and RET Somatic Mutation Profile

**Figure 3:**
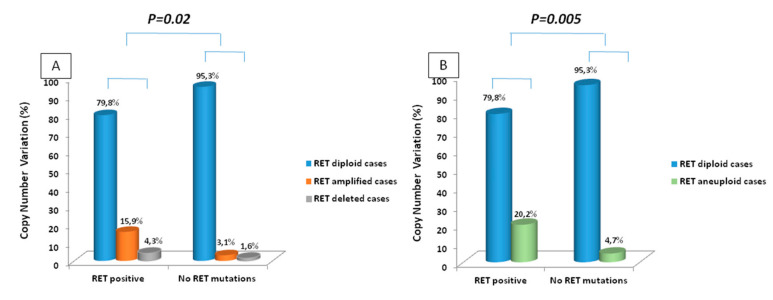
Comparison between the *RET* somatic mutation profile and the status of *RET* ploidy. (**A**) amplified and deleted cases are considered separately; (**B**) *RET* deleted and amplified cases (i.e., aneuploid cases) have been considered as a single category. To verify if there was a specific correlation with the type of *RET* mutation and *RET* gen CNV we classified our *RET* positive cases based on the *RET* categories of risk indicated by the ATA guidelines (highest, high and moderate) [11] and we found 57 cases with a *RET* mutation in the highest group, 23 cases in the high group and 14 cases in the moderate group. As shown in Figure 4, there was not a statistically significant correlation between *RET* ploidy and type of *RET* mutation neither if considering separately deleted, diploid, and amplified tumors (Figure 4A) or when we considered aneuploid (i.e., deleted+amplified) vs. diploid tumors (Figure 4B).

We used data on *RET* CNV generated by the in silico method to study the correlation between the *RET* gene CNV and the somatic mutation profile. In the whole series, 94 out of 158 (59.5%) cases had *RET* gene somatic mutation, 64 in 158 (40.5%) were either positive for *RAS* mutations (*n* = 37) or negative for any genetic alteration (*n* = 27). As shown in Figure 3A, the prevalence of *RET* amplified and deleted cases was significantly higher (*p* = 0.02) in the group of *RET* positive tumors (15 in 94 (15.9%) amplified; 4 in 94 (4.2%) deleted and 75 in 94 (79.9%) diploid) than in the *RET* negative/*RAS* positive tumors (2 in 64 (3.1%) amplified; one in 64 (1.6%) deleted and 61 in 64 (95.3%) diploid). In particular, *RET/RAS* negative cases did not show any *RET* gene CNV and the only three cases with *RET* gene CNV were positive for *RAS* mutations. This difference was even more statistically significant (*p* = 0.005) when we analyzed *RET* deleted and amplified cases together (i.e., aneuploid cases) vs. *RET* diploid cases (Figure 3B).

### 3.3. Correlation between RET Gene CNV and Variant Allele Frequency (VAF)

**Figure 5:**
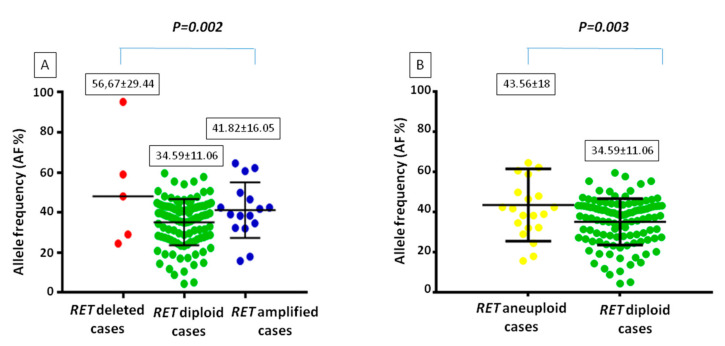
Comparison between the allelic frequency of *RET* somatic mutation and the status of *RET* ploidy. (**A**) amplified and deleted cases are considered separately; (**B**) *RET* deleted and amplified cases (i.e., aneuploid cases) have been considered as a single category.

CNV of the *RET* gene was then correlated with the allelic frequency of the *RET* somatic mutations. As shown in Figure 5A, VAF is much higher in deleted (56.67± 29.44) and amplified (41.82 ± 16.05) cases compared to diploid samples (34.59 ± 11.06) and this difference was statistically significant (*p* = 0.002). The difference in the *RET* mutation VAF remains statistically significant (*p* = 0.003) when analyzing aneuploid (i.e., amplified and deleted, 43.56 ± 18) vs. diploid cases (34.59 ± 11.06) (Figure 5B).

### 3.4. Correlation between Outcome and Ploidy Status

Among the 158 patients included in the study, clinical data regarding the outcome were available in 142 patients: 83 in 142 (58.4%) were disease-free or had a biochemical persistence of the disease, while 59 in 142 (41.6%) were dead or had a metastatic persistence of the disease. Although the difference was not statistically significant (*p* = 0.23), the prevalence of patients with metastatic disease was higher in the groups of *RET* amplified or deleted cases while in diploid cases a higher prevalence of disease-free patients was observed. Indeed, a statistically significant difference was found when comparing the ploidy status, either separately (*p* = 0.03) or when combining amplified and deleted cases (*p* = 0.02), and mortality with a highest prevalence of dead patients in the group of cases with a *RET* gene CNV. In particular, the statistically significant difference was confirmed when the analysis was restricted to the group of *RET* positive cases (*p* = 0.02).

**Figure 6:**
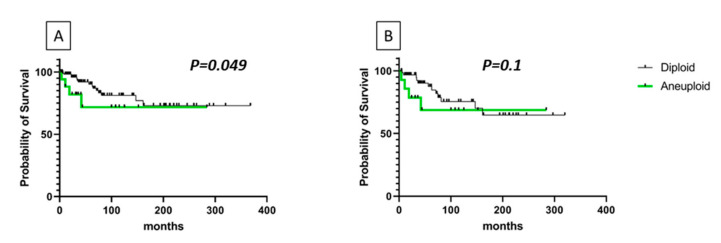
Survival curves of diploid or aneuploid *RET* MTC patients. (**A**) patients with an altered number of *RET* copies showed a significantly (*p* = 0.049, by Gehan-Breslow-Wilcoxon test) lower survival. (**B**) *RET* positive patients with a *RET* gene CNV showed a lower survival with respect to *RET* positive patients with a diploid status (*p* = NS).

We then evaluated the overall survival of our groups of patients. As shown in Figure 6A, patients with a *RET* gene CNV (aneuploid) showed a lower survival (*p* = 0.049) with respect to patients with a diploid *RET* gene. Among aneuploid cases those with a *RET* gene deletion were found to have the lowest survival. When we restricted the analysis to the *RET* positive cases, although we lost the statical significance, patients with a *RET* gene CNV had a lower survival with respect to *RET* positive patients with a diploid status (Figure 6B).

## 4. Discussion

Somatic CNV has been found in several types of carcinomas [12,13,14] and, in some of them, they are known to be drivers of cancer development [15] and progression [16]. They are supposed to be drivers also in PTC since they have been found in a subgroup without any other driver mutation [10]. Also, MTC is characterized by CNV that correlate with *RET* mutation, particularly with *RET* M918T [17]. However, only one study from our group [9] concentrated the attention on the specific CNV of the *RET* gene and/or chromosome 10 to verify if it could represent an alternative method of *RET* activation. Because the last 10 years have been characterized by the introduction of advanced methodologies in molecular biology analysis, we performed this study in a bigger group and with a new methodology as to better define this potential role.

There are some human tumors characterized by high genomic instability such as melanomas [18]. These tumors are very aggressive, and it has been demonstrated that cases with poor prognosis are associated with a significantly higher incidence of genomic imbalance [19,20]. In the present study, the majority of samples (86.7%) are diploid for the *RET* gene suggesting substantial genomic stability of this thyroid cancer histotype. The genomic stability of MTC was already appreciated in previous studies of our group showing that only 8% of sporadic MTC showed more than one somatic mutation and that only 20% of cases have different mutational profiles when comparing the primary with the corresponding metastatic tissues [21,22]. Supporting our data, Fisk et al. also found a few genomic alterations when analysing sporadic MTC by array-CGH [17].

Among the aneuploidies, although infrequent, the most encountered phenomenon is that of *RET* amplification (10.7%) while only 3.2% of the samples have a loss of *RET* gene. In agreement with our results, amplifications of the *RET* gene have been previously described in both sporadic and hereditary cases [9,23,24] while no data are present in the literature on the *RET* loss of copy number. The phenomenon of copy number gain of oncogenes is largely reported and correlated with an increase of gene expression that ultimately results in the development of the neoplastic process [25]. At variance, copy number loss is mainly reported for tumor suppressor gene [26,27,28,29,30] and the meaning of copy number loss for oncogenes, as well as for *RET*, remains to be clarified. Nevertheless, the role of *RET* as a tumor suppressor gene has been proven in lung cancer [31] and involved in the progression of colon adenomas to cancer. No data are now available on the potential role of *RET* as a tumor suppressor gene in thyroid carcinomas and the phenomenon of its deletion is not easy to be explained. However, although our methodology does not allow to identify which allele, either the mutated or the wild type, is amplified or deleted one possible explanation it could be that the mutated allele is amplified in tumors defined as “amplified”. This hypothesis is supported by the evidence that in other human tumors the mutated allele is also amplified (i.e., EGFR in lung cancer) [32]. At variance, the wild type allele might be lost in tumors defined as “deleted”. This hypothesis is supported by the evidence that the allelic frequency of the *RET* somatic mutations is higher in “deleted tumors” than in “amplified” or “diploid” cases.

Regarding the potential driver role of *RET* aneuploidy, particularly gene amplification, since the prevalence of *RET* amplified and deleted cases is higher in the group of *RET* positive tumors, we can define that *RET* gene CNV could not be considered as an alternative mechanism of *RET* activation but rather it could play a potentiating role in the progression of MTC. As previously suggested [33] aneuploidy and in particular CNV could occur early in the process of tumoral transformation, likely due to carcinogens exposition conferring instability to the cell genome and later to the occurrence of additional mutations, for example, *RET* somatic mutations [24].

The mechanism by which genomic DNA regions are amplified is still unclear: to understand whether the different aggressiveness of the mutations could play a role in the formation of the CNV, we classified our *RET* positive cases based on the risk categories indicated by the ATA guidelines (highest, high and moderate) [11]. We observed that there was no correlation between *RET* ploidy and type of *RET* mutation, suggesting that the type of somatic mutation does not play a role in CNV, but that probably the driver mutation promoting the neoplastic transformation of the cell, also provokes a destabilization of the cell that involves the molecular mechanisms at the basis of aneuploidy.

One of the goals of this study was to understand if *RET* gene CNV could play a role in disease progression and to clarify if the presence of *RET* altered copies could correlate with a worse outcome. Several authors [34,35,36] suggest that the CNV burden correlated with a poor prognosis. We have observed that most disease-free patients fall into the diploid group and that, on the contrary, most patients with persistent disease are included in the category of aneuploidy. Although this trend was not statistically significant, the presence of *RET* gene CNV is significantly correlated with mortality and with a lower rate of survival, thus suggesting it to be a prognostic factor of bad prognosis, even when we limited the analysis to the *RET* positive group, thus suggesting that *RET* gene CNV can be considered an additional prognostic factor of mortality independent from the presence of *RET* somatic alterations.

## 5. Conclusions

In conclusion, *RET* gene CNV is a rare event in sporadic MTC and mainly associated with *RET* somatic mutation, thus countering the hypothesis that *RET* gene CNV could be a driver mechanism of tumoral transformation in non-mutated MTC. There is still uncertainty about which *RET* allele is amplified or deleted, but in both cases, the correlation with a higher allelic frequency can be theoretically justified with the loss of the wild type allele in case of deletion and gain of the mutated allele in case of amplification. Finally, we found a positive correlation between *RET* gene CNV and a worse outcome of MTC patients, particularly with the death disease correlated.

## Figures and Tables

**Figure 4:**
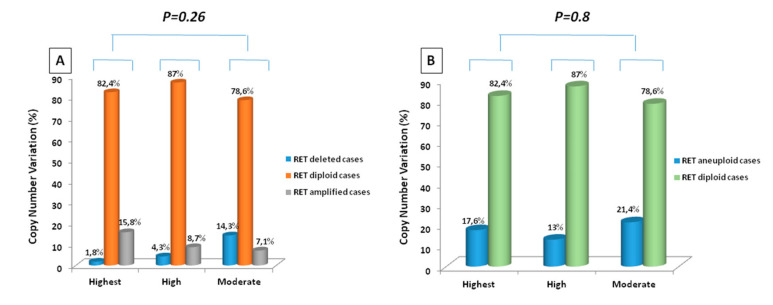
Comparison between the type of *RET* somatic mutation classified according to the ATA guidelines and the status of *RET* ploidy. (**A**) amplified and deleted cases are considered separately; (**B**) *RET* deleted and amplified cases (i.e., aneuploid cases) have been considered as a single category.

**Table 1:** Identification of deleted, diploid and amplified cases according to MLPA categories.

MLPA ASSAY n/tot (%)	PLOIDY CALL
DQ < 0.8 3/78 (3.8)	deleted
0.8 ≤ DQ < 1.3 66/78 (84.6)	diploid
DQ ≥ 1.3 9/78 (11.6)	amplified

**Table 2:** Identification of deleted, diploid and amplified cases according to Ion reporter CNV categories.

ION REPORTER n/tot (%)	PLOIDY CALL
CNV < 0.7 5/158 (3.2)	deleted
0.7 < CNV < 1.4 137/158 (86.7)	diploid
CNV > 1.4 16/158 (10.7)	amplified

## Data Availability

The data presented in this study are available on request from the corresponding author.

## References

[1] Chmielik E., Rusinek D., Oczko-Wojciechowska M., Jarzab M., Krajewska J., Czarniecka A., Jarzab B. (2018). Heterogeneity of Thyroid Cancer. Pathobiology.

[2] Elisei R., Tacito A., Ramone T., Ciampi R., Bottici V., Cappagli V., Viola D., Matrone A., Lorusso L., Valerio L. (2019). Twenty-five years experience on RET genetic screening on hereditary MTC: An update on the prevalence of germline RET mutations. Genes.

[3] Romei C., Cosci B., Renzini G., Bottici V., Molinaro E., Agate L., Passannanti P., Viola D., Biagini A., Basolo F. (2011). RET genetic screening of sporadic medullary thyroid cancer (MTC) allows the preclinical diagnosis of unsuspected gene carriers and the identification of a relevant percentage of hidden familial MTC (FMTC). Clin. Endocrinol..

[4] Agrawal N., Jiao Y., Sausen M., Leary R., Bettegowda C., Roberts N.J., Bhan S., Ho A.S., Khan Z., Bishop J. (2013). Exomic sequencing of medullary thyroid cancer reveals dominant and mutually exclusive oncogenic mutations in RET and RAS. J. Clin. Endocrinol. Metab..

[5] Ciampi R., Romei C., Ramone T., Prete A., Tacito A., Cappagli V., Bottici V., Viola D., Torregrossa L., Ugolini C. (2019). Genetic Landscape of Somatic Mutations in a Large Cohort of Sporadic Medullary Thyroid Carcinomas Studied by Next-Generation Targeted Sequencing. iScience.

[6] Heilmann A.M., Subbiah V., Wang K., Sun J.X., Elvin J.A., Chmielecki J., Sherman S.I., Murthy R., Busaidy N.L., Subbiah I. (2016). Comprehensive Genomic Profiling of Clinically Advanced Medullary Thyroid Carcinoma. Oncology.

[7] Simbolo M., Mian C., Barollo S., Fassan M., Mafficini A., Neves D., Scardoni M., Pennelli G., Rugge M., Pelizzo M.R. (2014). High-throughput mutation profiling improves diagnostic stratification of sporadic medullary thyroid carcinomas. Virchows Arch..

[8] Ramone T., Romei C., Ciampi R., Tacito A., Piaggi P., Torregrossa L., Ugolini C., Elisei R. (2019). Differential expression of RET isoforms in normal thyroid tissues, papillary and medullary thyroid carcinomas. Endocrine.

[9] Ciampi R., Romei C., Cosci B., Vivaldi A., Bottici V., Renzini G., Ugolini C., Tacito A., Basolo F., Pinchera A. (2012). Chromosome 10 and RET gene copy number alterations in hereditary and sporadic Medullary Thyroid Carcinoma. Mol. Cell. Endocrinol..

[10] Agrawal N., Akbani R., Aksoy A., Ally A., Arachchi H., Asa S.L., Auman J.T., Balasundaram M., Balu S., Stephen B. (2014). Integrated Genomic Characterization of Papillary Thyroid. Cell.

[11] Kloos R.T., Eng C., Evans D.B., Francis G.L., Gagel R.F., Gharib H., Moley J.F., Pacini F., Ringel M.D., Schlumberger M. (2009). Medullary thyroid cancer: Management guidelines of the American Thyroid Association. Thyroid.

[12] Jabs V., Edlund K., König H., Grinberg M., Madjar K., Rahnenführer J., Ekman S., Bergkvist M., Holmberg L., Ickstadt K. (2017). Integrative analysis of genome-wide gene copy number changes and gene expression in non-small cell lung cancer. PLoS ONE.

[13] Ried T., Meijer G.A., Harrison D.J., Grech G., Franch-Expósito S., Briffa R., Carvalho B., Camps J. (2019). The landscape of genomic copy number alterations in colorectal cancer and their consequences on gene expression levels and disease outcome. Mol. Asp. Med..

[14] Zhang S.Q., Pan X.Y., Zeng T., Guo W., Gan Z., Zhang Y.H., Chen L., Zhang Y.H., Huang T., Cai Y.D. (2019). Copy Number Variation Pattern for Discriminating MACROD2 States of Colorectal Cancer Subtypes. Front. Bioeng. Biotechnol..

[15] Gonçalves E., Fragoulis A., Garcia-Alonso L., Cramer T., Saez-Rodriguez J., Beltrao P. (2017). Widespread Post-transcriptional Attenuation of Genomic Copy-Number Variation in Cancer. Cell Syst..

[16] Hastings P.J., Ira G., Lupski J.R. (2009). A microhomology-mediated break-induced replication model for the origin of human copy number variation. PLoS Genet..

[17] Frisk T., Zedenius J., Lundberg J., Wallin G., Kytölä S., Larsson C. (2001). CGH alterations in medullary thyroid carcinomas in relation to the RET M918T mutation and clinical outcome. Int. J. Oncol..

[18] Martincorena I., Campbell P.J. (2015). Somatic mutation in cancer and normal cells. Science.

[19] Kaufmann W.K., Carson C.C., Omolo B., Filgo A.J., Sambade M.J., Simpson D.A., Shields J.M., Ibrahim J.G., Thomas N.E. (2014). Mechanisms of chromosomal instability in melanoma. Environ. Mol. Mutagen..

[20] Hirsch D., Kemmerling R., Davis S., Camps J., Meltzer P.S., Gaiser T., Mannheim M.F. (2015). Poor Outcome in Malignant Melanoma. Cancer Res..

[21] Romei C., Ciampi R., Casella F., Tacito A., Torregrossa L., Ugolini C., Basolo F., Materazzi G., Vitti P., Elisei R. (2018). RET mutation heterogeneity in primary advanced medullary thyroid cancers and their metastases. Oncotarget.

[22] Romei C., Casella F., Tacito A., Bottici V., Valerio L., Viola D., Cappagli V., Matrone A., Ciampi R., Piaggi P. (2016). New insights in the molecular signature of advanced medullary thyroid cancer: Evidence of a bad outcome of cases with double RET mutations. J. Med. Genet..

[23] Koch C.A., Huang S.C., Moley J.F., Azumi N., Chrousos G.P., Gagel R.F., Zhuang Z., Pacak K., Vortmeyer A.O. (2001). Allelic imbalance of the mutant and wild-type RET allele in MEN 2A-associated medullary thyroid carcinoma. Oncogene.

[24] Huang S.C., Torres-Cruz J., Pack S.D., Koch C.A., Vortmeyer A.O., Mannan P., Lubensky I.A., Gagel R.F., Zhuang Z. (2003). Amplification and overexpression of mutant RET in multiple endocrine neoplasia type 2-associated medullary thyroid carcinoma. J. Clin. Endocrinol. Metab..

[25] Wee Y.K., Wang T.F., Liu Y., Li X., Zhao M. (2018). A pan-cancer study of copy number gain and up-regulation in human oncogenes. Life Sci..

[26] Cheung M., Testa J.R. (2017). BAP1, a tumor suppressor gene driving malignant mesothelioma. Transl. Lung Cancer Res..

[27] Kresse S.H., Ohnstad H.O., Paulsen E.B., Bjerkehagen B., Szuhai K., Serra M., Schaefer K.-L., Myklebost O., Meza-Zepeda L.A. (2009). LSAMP, a novel candidate tumor suppressor gene in human osteosarcomas, identified by array comparative genomic hybridization. Genes. Chromosomes Cancer.

[28] Jia P., Zhao Z. (2019). Characterization of Tumor-Suppressor Gene Inactivation Events in 33 Cancer Types. Cell Rep..

[29] Hollander M.C., Blumenthal G.M., Dennis P.A. (2011). PTEN loss in the continuum of common cancers, rare syndromes and mouse models. Nat. Rev. Cancer.

[30] Levine A.J., Momand J., Finlay C.A. (1991). The p53 tumour suppressor gene. Nature.

[31] Luo Y., Kaz A.M., Kanngurn S., Welsch P., Morris S.M., Wang J., Lutterbaugh J.D., Markowitz S.D., Grady W.M. (2013). NTRK3 Is a Potential Tumor Suppressor Gene Commonly Inactivated by Epigenetic Mechanisms in Colorectal Cancer. PLoS Genet..

[32] Sholl L.M., Yeap B.Y., Iafrate A.J., Holmes-Tisch A.J., Chou Y.P., Wu M.T., Goan Y.G., Su L., Benedettini E., Yu J. (2009). Lung adenocarcinoma with EGFR amplification has distinct clinicopathologic and molecular features in never-smokers. Cancer Res..

[33] Li R., Yerganian G., Duesberg P., Kraemer A., Willer A., Rausch C., Hehlmann R. (1997). Aneuploidy correlated 100% with chemical transformation of Chinese hamster cells. Proc. Natl. Acad. Sci. USA.

[34] Taylor B.S., Schultz N., Hieronymus H., Gopalan A., Xiao Y., Carver B.S., Arora V.K., Kaushik P., Cerami E., Reva B. (2010). Integrative Genomic Profiling of Human Prostate Cancer. Cancer Cell.

[35] Hieronymus H., Schultz N., Gopalan A., Carver B.S., Chang M.T., Xiao Y., Heguy A., Huberman K., Bernstein M., Assel M. (2014). Copy number alteration burden predicts prostate cancer relapse. Proc. Natl. Acad. Sci. USA.

[36] Camacho N., Van Loo P., Edwards S., Kay J.D., Matthews L., Haase K., Clark J., Dennis N., Thomas S., Kremeyer B. (2017). Appraising the relevance of DNA copy number loss and gain in prostate cancer using whole genome DNA sequence data. PLoS Genet..

